# Bonding of Clear Aligner Composite Attachments to Ceramic Materials: An In Vitro Study

**DOI:** 10.3390/ma15124145

**Published:** 2022-06-10

**Authors:** Bashair A. Alsaud, Maher S. Hajjaj, Ahmad I. Masoud, Ensanya A. Abou Neel, Dalia A. Abuelenain, Amal I. Linjawi

**Affiliations:** 1 Department of Orthodontics, Faculty of Dentistry, King Abdulaziz University, Jeddah 21589, Saudi Arabia; bashair_alsaud@hotmail.com (B.A.A.); aemasoud@kau.edu.sa (A.I.M.); 2 Department of Restorative Dentistry, Faculty of Dentistry, King Abdulaziz University, Jeddah 21589, Saudi Arabia; mhajjaj@kau.edu.sa (M.S.H.); dabualenain@kau.edu.sa (D.A.A.); 3 Department of Preventive and Restorative Dentistry, College of Dental Medicine, University of Sharjah, Sharjah 27272, United Arab Emirates; eabouneel@sharjah.ac.ae; 4 UCL Eastman Dental Institute, Biomaterials & Tissue Engineering, Royal Free Hospital, Rowland Hill Street, London NW3 2QG, UK

**Keywords:** attachments, ceramics, clear aligner, composite, in vitro, orthodontics, surface conditioning

## Abstract

Background: We aim to evaluate the effect of surface conditioning, bonding agents and composite types on surface roughness (SR) and shear bond strength (SBS) of clear aligner composite attachments bonded to ceramics. Methods: One hundred and eighty IPS e.max CAD specimens were prepared. For SR, 60 specimens were divided according to surface conditioning (*n* = 15) into four groups: control, 9.6% hydrofluoric acid (HFA), 37% phosphoric acid (PhA), air abrasion (AA). SR was measured using a Profilometer and Atomic Force Microscopy. For SBS, 120 specimens were divided according to conditioning methods (*n* = 40) (9.6% HFA and 37% PhA or AA), then according to bonding agents (*n* = 20) (Assure universal bond (AUB) or Single bond universal (SBU)) and then according to composite type (*n* = 10): Filtek™ Z350 and Filtek™ Z350 XT flowable composite. SBS was measured using Instron testing machine. Descriptive and group comparison were calculated (*p* < 0.05). Results: AA had the highest SR, while the control had the lowest SR (*p* < 0.05). HFA had the highest, but insignificant SBS, followed by AA (*p* > 0.05). AUB had higher SBS than SBU (*p* < 0.001). Filtek™ Z350 produced higher SBS than Filtek™ Z350 XT flowable composite (*p* < 0.01). Conclusion: The combination of AA, AUB, and Filtek Z350 produced the highest SBS, followed by HFA, AUB, and Filtek Z350.

## 1. Introduction

Removable clear aligners have become increasingly popular, especially with increasingly aesthetic demands [1,2,3,4,5]. Clear aligners were introduced by Align Technology in 1998 as a series of clear, removable, thermoplastic appliances to be worn by the patient in a sequence to obtain the proper targeted results [6,7]. Compared to other removable appliances, some tooth movements such as extrusion, rotation and root control were difficult to control using aligners, which has led some companies to introduce buttons made of composite resins known as “attachments”. Attachments are placed on tooth surfaces as retentive elements to expand the field of the aligner to control movements that would be difficult or cannot be achieved without them [5,8,9].

Adult orthodontic treatment using clear aligners is also increasing in demands. However, multiple teeth restorations, including ceramic crown and bridges in those age group, create some challenges to the orthodontists. Recently, lithium disilicate became the most popular material for crowns that offer undoubtful advantages [10,11]. Manufacturing technologies allow it to have both high performance and high aesthetic potential, as well as excellent consistency when it comes to precision and accuracy and short production times [11]. Moreover, lithium disilicate is a translucent material that does not require the use of veneering porcelain, which greatly reduces the chances of porcelain chipping off. Furthermore, the ceramic surface is capable of acid etching and chemical bonding through the application of silane coupling agent and resin cement [12]. Lithium disilicate and other high-crystalline content ceramics pose a significant challenge for orthodontic attachment bonding [13]. They consist of amorphous matrix containing 60–70% crystalline phase, which cause them to be more resistant to acid etching than feldspathic ceramics [14,15]. Without roughening the ceramic’s surface, composite application to the ceramic surface will result in a bond that is too weak for orthodontic tooth movement [16]. Therefore, various surface conditioning options (mechanical or chemical) to achieve durable bond strength have been advocated. Mechanical roughening can be performed by air abrasion or using a diamond bur. On the other hand, chemical roughening includes the application of hydrofluoric acid (HFA) and phosphoric acid (PhA), followed by silane coupling agents [16,17,18,19]. Sundfeld et al. and Garboza et al. showed that etching lithium disilicate ceramics with 10% HFA for 20 s with subsequent silane application resulted in high bond strengths [20,21]. Moreover, Al Rifaiy in 2018 [22] and Alqerban in 2019 [23] found that the highest shear bond strength (SBS) was obtained by increasing the etching time of 9.5% HFA to 90 s and 60 s, respectively. In 2020, Souza et al. compared different HFA etching times (20, 60 and 120 s + silanization or Scotch Bond Universal) on the bond strength of lithium disilicate. They found that etching with HFA for 60 s followed by silane produced the highest SBS but they were not significantly different from 20 or 120 s. Moreover, Scotchbond Universal compromised the SBS except for applying HFA for 120 s [24]. On the other hand, Mehmeti et al., in 2019, compared the effect of 5% HFA etching and 37% PhA etching for 120 s and found that they both provided similar bond strengths [25].

Currently, with the availability of multiple types of aesthetic restorations and bonding techniques, orthodontists are facing challenges in determining the best methods in surface conditioning and bonding procedures to ceramics that makes a good attachments bond and that does not affect the ceramic’s surface after debonding [13,19]. Moreover, choosing the best composite type to produce durable attachments is considered another challenge. Moreover, the majority of studies have been conducted on orthodontic brackets bonded to feldspathic ceramic; however, studies on other types of ceramics are limited [19]. Thus, the aim of this study is to evaluate the effect of three different factors (surface conditioning methods, bonding agents and composite types) on the shear bond strength (SBS) of composite attachments bonded to lithium disilicate ceramics. The study also aims to evaluate the effect of different surface conditioning methods on the surface roughness of lithium disilicate ceramic material.

## 2. Materials and Methods

This is an in vitro laboratory study carried out after obtaining ethical approval from the Research Ethics Committee at the Faculty of Dentistry, King Abdulaziz University, Jeddah, Saudi Arabia (Ethical # 36-04-2020).

### 2.1. Materials

Table 1 lists the materials, chemical compositions and manufacturer used in this study.

### 2.2. Sample Size Calculation

Based on previous research [19] and the power of test calculation (power analysis) using α level of 0.05 and 80% power and effect size equal to 0.7 for the shear bond strength, the sample size was found to be 6 samples per group. However, 10 samples per group was included in this study.

### 2.3. Ceramic Specimen Preparation

Lithium disilicate (IPS e-max) was sliced into smaller rectangular slices under water cooling using a diamond saw wafering blade mounted on an Isomet machine (Isomet 5000; Buehler, Lake Bluff, IL, USA) to produce 180 samples with the dimensions of 15 × 7.5 × 2 mm^3^. The surface of each sample was then smoothed using sandpaper with #220, 600 and 1200 grits, and the blocks underwent crystallization in a furnace (Ivoclar vivadent, Schaan, Liechtenstein) using the IPS e.max CAD crystallization parameters following the manufacturer’s recommendation. A layer of VITA Akzent glaze (VITA Akzent glaze, Vita Zahnfabrik, Seckingen, Germany) was then applied to the dull surfaces, and the samples were heated in the furnace sintering machine (Ivoclar vivadent, Schaan, Liechtenstein) as recommended by the manufacturer. After that, silicone mold was fabricated, and the blocks were embedded in chemically activated acrylic resin (JET, Dental Articles Classic, Sao Paulo, Brazil). All ceramic blocks were cleaned ultrasonically in distilled water for 5 min using the power sonic 405 device (Power Sonic 405, Whashin Co., Seoul, Korea). Then, the blocks were left for air drying on a piece of gauze for 10 min.

### 2.4. Surface Roughness Test

A total of 60 samples were divided according to the surface conditioning method into four groups (*n* = 15): Group I: Control/no treatment; Group II: Etching with 9.6% HFA for 1 min; Group III: Etching with 37% PhA for 2 min; Group IV: Air abrasion (AA) using 50 μm AL_2_O_3_ for 5 s under 2 bar pressures in a perpendicular direction to the ceramic block surface and at 10 mm distance using sandblaster unit (Duostar Z2, Bego, Bremen, Germany).

All samples were then cleaned ultrasonically and dried as described above. Surface roughness was measured using Bruker Optical Profilometer (Contour GT-K, Bruker, Tucson, AZ, USA) and Atomic Force Microscope (AFM) (Bruker NH-2, 1041, Tucson, AZ, USA). The AFM images were generated with a slow scan rate (1 Hz), scan size of 20 × 20 μm and a scanning head size of 10 μm.

### 2.5. Shear Bond Strength (SBS) Test

**Figure 1:**
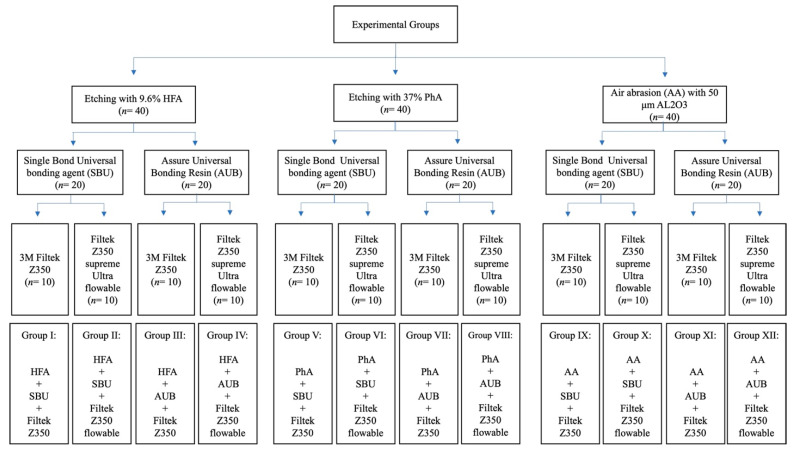
SBS experimental flowchart and groups.

A total of 120 specimens were divided according to the surface treatment method into three groups (*n* = 40). Each group was subsequently divided according to the adhesive type (*n* = 20) and composite type (*n* = 10), as shown in Figure 1.

After surface treatments, bonding adhesives were applied. For the SBU adhesive, one coat of the adhesive was applied by a light hand scrubbing motion using a micro brush (Dentsply, New York, NY, USA) on the ceramic’s surfaces for 20 s, air dried for 5 s and finally light cured for 10 s, as recommended by the manufacturer. For the AUB adhesive, the porcelain conditioner was applied for 2 min and then dried for 30 s. One coat of ABU was then applied, air dried for 5 s and light cured for 10 s, as recommended by the manufacturer.

**Figure 2:**
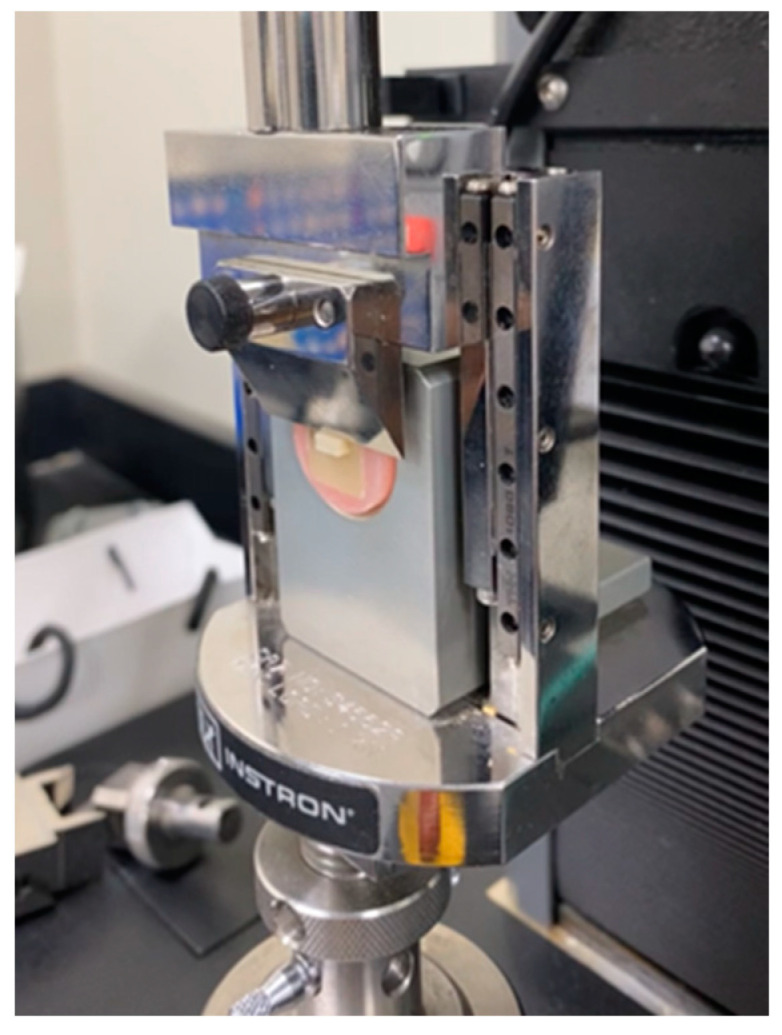
Shear bond strength testing using an Instron testing machine.

A rectangular acrylic mold was constructed with dimensions (5 × 2 × 1.5 mm^3^) to simulate the dimensions of the rectangular attachments recommended by the Invisalign company (Invisalign, Align Technology, Santa Clara, CA, USA) and was used to build the composite attachments. The mold was laid on a glass slab, filled with composite resins that were light cured for 20 s, as recommended by the manufacturers and then removed. All ceramic blocks with bonded composite attachments were subjected to 10,000 cycles of alternate 30 s baths at 5 °C and 55 °C, with a 5 s interval between immersions using a thermocycler (SD Mechatronik Thermocycler, SD Mechatronik GMBH, Westerham, Germany). After thermocycling, all specimens were loaded until failure under 50 Kg and 0.5 mm/min using a universal testing machine (ElectroPlus E1000, Instron, Canton, MA, USA) (Figure 2). The SBS value was calculated in MPa.

### 2.6. Statistical Analysis

Descriptive statistics including the mean and standard deviation (SD) for the surface roughness and SBS. Multiple-ANOVA and post hoc Tukey tests were used for surface roughness. For the shear bond strength, T-test and multiple-ANOVA analysis with post hoc Tukey tests for group comparisons were carried out. A significance level of 0.05 was set for all analysis.

## 3. Results

### 3.1. Surface Roughness

**Figure 3:**
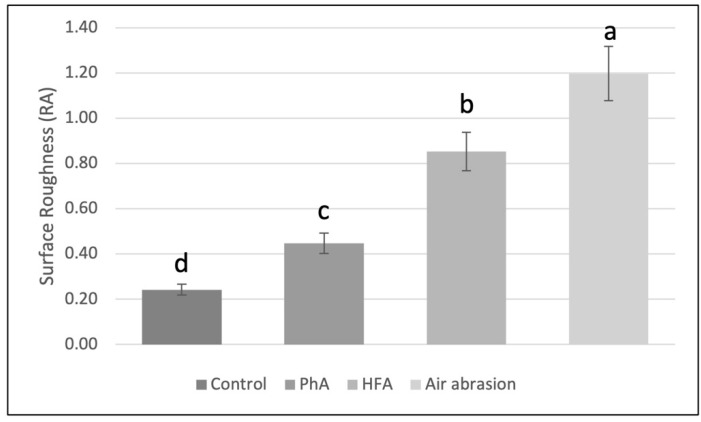
Ceramic surface roughness results. Values with different superscript letters indicate significant differences (*p* < 0.05) (a > b > c > d).

The lowest mean surface roughness was obtained for the control group (0.24 ± 0.08 μm), while air abrasion had the highest mean surface roughness (1.20 ± 0.30 μm). There was a statistically significant difference between all tested groups (*p* < 0.05), as shown in Figure 3.

**Figure 4:**
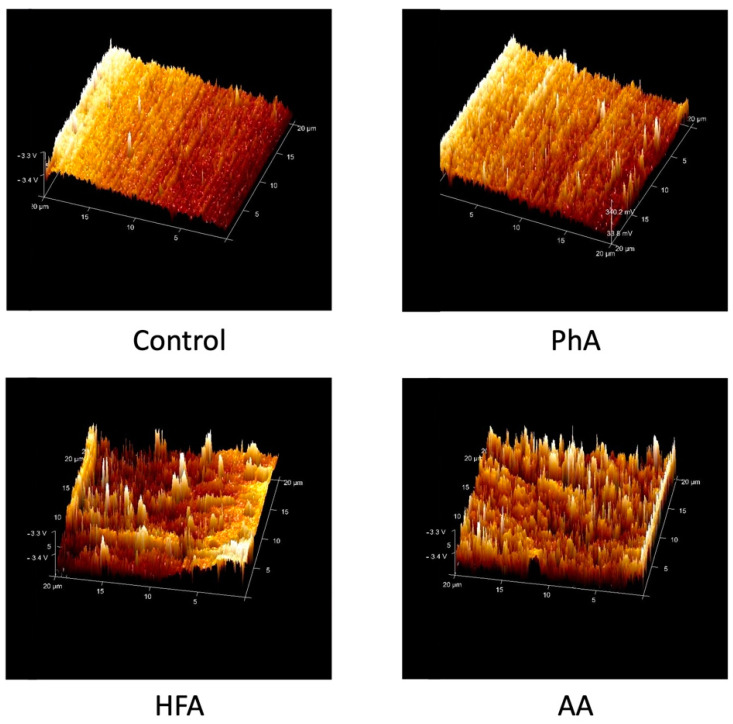
AFM representative images for the surface roughness of the ceramic surfaces treated with different conditioning methods; control, PhA etching group; HFA etching group, AA group.

Representative AFM images of the ceramic surfaces treated with different conditioning methods are presented in Figure 4. The control group showed the smoothest surface, followed by PhA etching, which had little small projections followed by HFA etching which produced a non-uniform pattern and distinct projections as sharp spikes. Air abrasion conditioning method showed the roughest surface with multiple irregularities and longer sharp spikes than the other groups.

### 3.2. Shear Bond Strength (SBS)

Table 2 shows the SBS results of different conditioning methods, bonding agents and composite types. For the conditioning methods, HFA had the highest SBS with a mean value of (15.82 ± 4.72 MPa) followed by AA (14.91 ± 5.38 MPa), but there was no statistically significant difference between them (*p* > 0.05). On the other hand, PhA provided the lowest SBS value (5.22 ± 4.03 MPa), which was significantly different from the other groups (*p* < 0.0001).

The shear bond values for the different bonding agents showed that AUB resulted in statistically higher SBS than SBU (*p* = 0.001) with a mean strength of (14.04 ± 6.04 and 9.93 ± 6.80 Mpa), respectively.

For composite types, the results showed a statistically significant difference between the groups (*p* = 0.003). Filtek Z350 had higher SBS value than Filtek Z350 supreme Ultra flowable (13.79 ± 7.47 and 10.18 ± 5.38 MPa), respectively.

**Figure 5:**
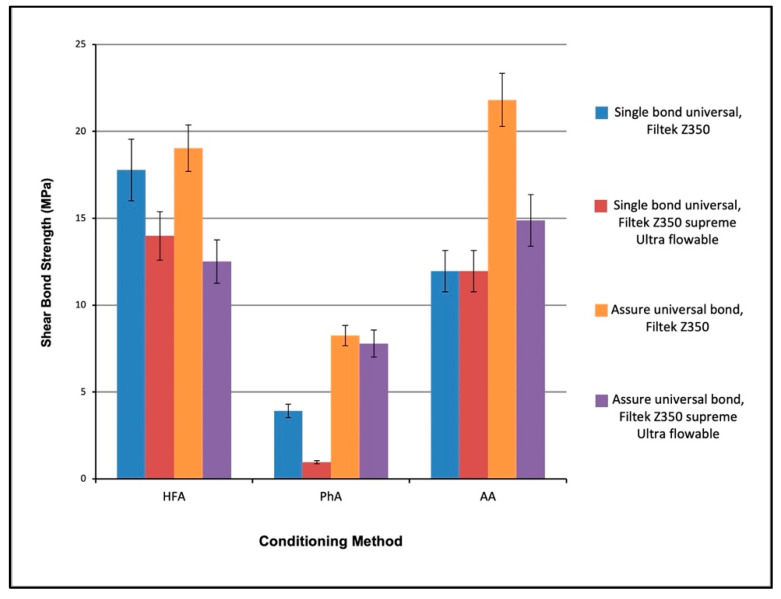
SBS for the interaction between different conditioning methods, bonding agents and composite types bonded to lithium disilicate glass ceramics. The interactions are presented grouped according to conditioning methods.

The interaction between different conditioning methods, bonding agents and composite types is presented in Figure 5. Three-way ANOVA revealed that there is a statistically significant difference between all groups. Tests of between-subjects’ effect were performed, and they showed that there is statistically significant difference between the surface conditioning methods (*p* < 0.0001), bonding agents (*p* < 0.0001) and composite types (*p* < 0.0001). Results also showed a statistically significant difference in the interaction of the surface conditioning methods with the bonding agents (*p* < 0.0001) and the interaction of the conditioning methods with the bonding agents and composite types (*p* = 0.02).

The post hoc Tukey test revealed that the highest SBS was gained by the interaction of AA with AUB and Filtek Z350 composite, followed by the interaction of HFA, AUB and Filtek Z350, and then the interaction of HFA, SBU and Filtek Z350 (21.80 ± 3.86; 19.03 ± 3.00; 17.77 ± 6.94 MPa), respectively. The interaction of PhA with SBU and Filtek Z350 supreme ultra-flowable showed the lowest strength value with (0.96 ± 1.86 MPa) (Table 3).

## 4. Discussion

Since ceramics are now used frequently in prosthodontics to meet patient aesthetic demands, orthodontists must choose proper methods of bonding to these materials to achieve optimum outcomes [26]. Ceramic materials are versatile and continuously evolving, making it possible to achieve the desired aesthetic results. In this study, lithium disilicate ceramics were chosen due to their superior aesthetic, optical and mechanical properties compared with the conventional feldspathic or leucite crowns [19]. It is available in a variety of shades and translucency levels according to the patient’s needs [11]. Mechanically, it has high flexure strength and a fracture toughness [11] and accurate 2D and 3D marginal fittings [27]. Compared to CAD-CAM zirconia, CAD-CAM lithium disilicate had smaller marginal gaps [28] and is considered a suitable material even for subgingival restorations directly contacting the sulcular epithelial tissues because it showed the best biocompatibility when compared to zirconia and cobalt–chromium alloys [29].

When it comes to ceramic bonding, orthodontists have two conflicting goals: ensuring a superior bond strength during treatment to eliminate bonding failures and preserving the integrity of artificial restorations after debonding [17]. Chemical bonding and micromechanical interlocking are important in bonding to ceramics [30,31,32,33,34]. In clinical practice, different surface treatment techniques have been used to create a micromechanically retentive ceramic surface. As observed, air abrasion generated the highest surface roughness followed by HFA, while PhA and control groups showed the least surface roughness. This could be explained by the irregularities created by sandblasting, which resulted in the removal of glassy matrix and an increase in surface free energy with a consequent increase in the bond strength [35,36]. Moreover, the increase in surface roughness provides additional retention sites where adhesive cement can easily interlock to increase the shear bond strength [37]. The altered topography of the HFA could be attributed to the acid’s ability to attack the ceramics’ glassy matrix, which leads to the exposure of lithium disilicate crystals; hence, surface roughness and irregularities increased, which will improve micromechanical interlocking abilities with resin cement [38,39]. PhA resulted in a low surface roughness, which might be due to the inability of the acid to remove the glassy matrix, which will lead to lower surface areas for retention. These results were confirmed by AFM images and were in line with previous studies [19,40,41]. Contrarily, Dilber et al. showed nearly similar results when it comes to air abrasion and control groups but lower roughness parameter of HFA, which might be due to the lower concentrations and etching time used (5% for 20 s only) [30]. Furthermore, Sudre et al. had higher roughness values for the control and HFA groups [42]. However, Dilber et al. and Sudre et al. used IPS Empress 2 [30,42].

AFM images of the ceramic surfaces revealed that air abrasion and HFA conditioning methods increased the surface roughness compared to PhA and untreated surfaces. Air abrasion produced the roughest irregular surface with multiple sharp projections. HFA resulted in a non-uniform surface with less peaks and valleys than air abrasion. PhA etching showed small surface changes compared to the untreated control group, which were observed as short spikes. The images support the profilometry results where air abrasion had the roughest surface followed by HFA, PhA and finally the control group.

When it comes to SBS between different conditioning methods, the results revealed that HFA produced the highest SBS value compared to air abrasion. However, there was no statistically significant difference between them. In contrast, PhA etching had the lowest strength value, which was significantly different from the other methods and lower than the recommended bond strength of 6–8 MPa, as established by Reynolds for bonding orthodontic brackets to natural teeth [43]. For bonding attachments to natural teeth, Chen et al. in 2020 found that the highest SBS was gained by bonding attachments with SonicFill composite (23.49 MPa). Filtek Z350XT had an SBS of 20.53 MPa while bonding with Filtek Z350XT Flowable resulted in 20.53 MPa [2]. The AFM images support these results since air abrasion and HFA had a rougher surface than PhA specimens. Roughening the surface provides additional potential retention sites where the adhesive cement can easily be interlocked, enhancing the shear bond strength [37].

In order to achieve proper bonding, micromechanical retention achieved by conditioning methods must be followed by silane coupling agents as a means of bonding inorganic ceramic surfaces with organic resins [20,44,45,46]. Since 1977, silane has been used as a bond enhancer [47]. Through the formation of a siloxane bond, it increases the ceramics surface energy, as well as the cement’s wettability. By doing so, microscopic interactions will occur between the two materials [44,48,49]. As “all-in-one” adhesives, universal bonding agents simplify the conventional bonding process and reduce chair times [50]. Silane-containing adhesives that bond silica-based ceramics such as single bond universal have been developed. In addition to their benefit in reducing the operation process, the adhesives’ manufacturers claim that these adhesives achieve excellent bonding properties and do not require silane treatment [50,51]. However, the effectiveness of universal adhesives containing silanes remains unclear [46,48]. In this study, the Assure universal bonding agent had a higher SBS value compared to single bond universal bonding agent. A possible explanation is that bonding strength decreases when silane and methacrylate monomers are present in one solution [52]. In addition, MDP, a type of monomer commonly found in universal adhesives, has been reported to promote the condensation of silanol groups in the presence of silane and neutralizes silane [52]. Another explanation is the presence of bisphenol A diglycidylmethacrylate (bis-GMA), which might interfere with the condensation between the silanol groups of silane and the OH groups of ceramics [52]. Moreover, the silane present in universal adhesives may also be insufficient [50]. This result agrees with previous studies [21,53].

There are several characteristics of the ideal attachment material, including its ease of use, resistance to wear and difficulty of fall off. Currently, there is constant progress in clinical research on clear aligner technology, whereas research into attachment material selection is still limited [2]. Based on viscosity, two commonly used composite resins for attachment bonding were used in this study. They are as follows: a low-viscosity flowable resin (Filtek Z350 supreme Ultra flowable composite) and a high-viscosity universal restorative material (Filtek Z350 composite). Results showed that Filtek Z350 had higher SBS value than Filtek Z350 supreme Ultra flowable. This result is similar to the findings reported by Chen et al. who evaluated the effect of the same composite types on the SBS of extracted premolars and found that the flowable composite showed lower bond strength (15.3 +/− 2.33 MPa) compared to the conventional Filtek Z350 (20.53 +/− 2.59 MpPa) [2]. It is likely that the amount of inorganic fillers played a major role in these results. Filtek Z350 contains 78.5% of inorganic fillers, while Filtek Z350 supreme Ultra flowable has 65% of inorganic fillers. By increasing inorganic filler content, polymerization shrinkage and stresses can be decreased resulting in a high bond strength. Consequently, a high SBS ensures a more stable treatment because attachments are less likely to fall off [2]. However, the SBS of both composites when bonded to natural teeth was higher than the SBS of both composites when bonded to lithium disilicate.

It is necessary to note, however, that there are some limitations of this study. For example, the experiments were conducted in vitro, so they may not represent clinical settings accurately. It is, therefore, necessary to confirm the results of the in vitro experiments with long-term clinical studies in the future. A rectangular acrylic mold was constructed for the composite attachments with the dimensions (5 × 2 × 1.5 mm^3^). Such dimensions are specific for this study since it was aimed to simulate the dimensions of the rectangular attachments recommended by the Invisalign company. It is similar to other studies assessing the clear aligner attachments [54]. Further studies are also needed to compare SBS between composite attachments bonded to natural tooth structure and ceramic materials.

## 5. Conclusions

Within the limitations of the present study, the following can be concluded:

1. AA caused a significant change in the ceramic’s surface microstructure and removed the glazed layer followed by HFA.
2. HFA conditioned group had the highest SBS value followed by AA. However, the difference between them was insignificant. On the other hand, the PhA group had the lowest SBS value, which was significantly different from the other groups (*p* < 0.05).
3. Two steps bonding agents such as AUB are more effective and provide significantly higher SBS values than SBU (*p* < 0.05).
4. Using Filtek Z350 composite in attachment bonding provides higher SBS than Filtek supreme Ultra flowable composite.
5. The combination of AA conditioning method with AUB and Filtek Z350 composite gave the highest SBS, followed by the combination of HFA etching with AUB and Filtek Z350.

The present study can help orthodontists to choose the proper materials when it comes to attachments bonding to lithium disilicate crowns and, hence, decreasing chair times by avoiding multiple bonding failures.

## Figures and Tables

**Table 1:** Materials used in this study with their chemical compositions and manufacturer name.

Material	Chemical Composition	Manufacturer
**Ceramic Material**		
Lithium disilicate glass ceramic (IPS e.max CAD)	Lithium meta-silicate crystals with approximately 40% crystals by volume. Additional contents: Li_2_O, K_2_O, MgO, AL_2_O_3_, P_2_O_2_	Ivoclar Vivadent, Schaan, Lichtenstein
**Surface conditioning methods**		
Phosphoric acid (PhA)	37% PhA, chlorhexidine digluconate, thickener, stain and water	Maquira
Hydrofluoric acid (HFA)	9.6% HFA, 5.3% Ethyl alcohol, thickening agent, dye and water	Pulpdent
Aluminum oxide particles (AL_2_O_3_)	Al_2_O_3_ 99.80%, SiO_2_ 0.023%, Fe_2_O_3_ 0.035%, TiO_2_ 0.006%, CaO 0.01%, Na_2_O 0.15%	Zhermack
**Bonding agents**		
Single bond universal adhesive (SBU)	MDP phosphate monomer, Dimethacrylate resins, HEMA, Vitrebond copolymer, filler, ethanol, water, initiators, silane.	3M ESPE
Assure universal bonding agent (AUB)	2-Hydroxyethyl Methacrylate 10–30%, BisGMA 10–30%, 4-Dimethylaminobenzoic Acid 1–5%	Reliance orthodontic
Porcelain conditioner (PC)	Ethanol/Denatured 190 PROOF 30–50%, 3-(Trimethoxysilyl) propyl-2-methyl-2-propenoic acid 1–5%, acetone, ACS grade 30–50%	Reliance orthodontic
**Composite types**		
3M Filtek™ Z350 XT composite	Matrix: Bis-GMA, UDMA, Bis-EMA Filler: Silica, zirconia nanoparticles (20 μm) (72.5 wt%/55.9 vol%)	3M ESPE
3M Filtek™ Z350 XT flowable composite	Matrix: Bis-GMA, TEGDMA, procrylat resin Filler: yetterbium trifluoride, silica, zirconium oxide (46 vol%/65 wt%)	3M ESPE

**Table 2:** Comparison of SBS between the groups according to the three different factors assessed: conditioning methods, bonding agents and composite types.

Groups		N	Mean (Mpa)	Std Dev	*p*-Value
Conditioning methods	HFA	40	15.82 ^a^	4.72	<0.0001 ***
	AA	40	14.91 ^a^	5.38	
	PhA	40	5.22 ^b^	4.03	
Bonding agent type	Assure universal bond	60	14.04	6.04	0.001 **
	Single bond universal	60	9.93	6.80	
Composite type	Filtek Z350	60	13.79	7.47	0.003 **
	Filtek Z350 supreme Ultra flowable	60	10.18	5.38	

Significance levels: ** *p* < 0.01, *** *p* < 0.001. Values with different superscript letters indicate significant differences between the different conditioning methods (a > b).

**Table 3:** Post hoc Tukey test showing the interaction between the three factors: conditioning methods, bonding agents and composite types (*p* < 0.05).

Interactions						
AA, AUB, Filtek Z350	A					
HFA, AUB, Filtek Z350	A	B				
HFA, SBU, Filtek Z350	A	B				
AA, AUB, Filtek Z350 flowable		B	C			
HFA, SBU, Filtek Z350 flowable		B	C			
HFA, AUB, Flowable			C	D		
AA, SBU, Filtek Z350			C	D		
AA, SBU, Filtek Z350 flowable			C	D		
PhA, AUB, Filtek Z350				D	E	
PhA, AUB, Filtek Z350 flowable				D	E	
PhA, SBU, Filtek Z350					E	F
PhA, SBU, Filtek Z350 flowable						F

Levels not connected by the same letter are significantly different (*p* < 0.05) (A > B > C > D > E > F).

## Data Availability

Data will be provided upon request.

## References

[1] Akhoundi M.A., Kamel M.R., Hashemi S.M., Imani M. (2011). Tensile bond strength of metal bracket bonding to glazed ceramic surfaces with different surface conditionings. J. Dent..

[2] Chen W., Qian L., Qian Y., Zhang Z., Wen X. (2021). Comparative study of three composite materials in bonding attachments for clear aligners. Orthod. Craniofac. Res..

[3] Rossini G., Parrini S., Castroflorio T., Deregibus A., Debernardi C.L. (2015). Efficacy of clear aligners in controlling orthodontic tooth movement: A systematic review. Angle Orthod..

[4] Rosvall M.D., Fields H.W., Ziuchkovski J., Rosenstiel S.F., Johnston W.M. (2009). Attractiveness, acceptability, and value of orthodontic appliances. Am. J. Orthod. Dentofac. Orthop..

[5] Weir T. (2017). Clear aligners in orthodontic treatment. Aust. Dent. J..

[6] Alajmi S., Shaban A., Al-Azemi R. (2020). Comparison of Short-Term Oral Impacts Experienced by Patients Treated with Invisalign or Conventional Fixed Orthodontic Appliances. Med. Princ. Pract..

[7] Beers A.C., Choi W., Pavlovskaia E. (2003). Computer-assisted treatment planning and analysis. Orthod. Craniofac. Res..

[8] Barreda G.J., Dzierewianko E.A., Muñoz K.A., Piccoli G.I. (2017). Surface wear of resin composites used for Invisalign^®^ attachments. Acta Odontol. Latinoam..

[9] Tamer I., Oztas E., Marsan G. (2019). Orthodontic treatment with clear aligners and the scientific reality behind their marketing: A literature review. Turk. J. Orthod..

[10] Makhija S.K., Lawson N.C., Gilbert G.H., Litaker M.S., McClelland J.A., Louis D.R., Gordan V.V., Pihlstrom D.J., Meyerowitz C., Mungia R. (2016). Dentist material selection for single-unit crowns: Findings from the National Dental Practice-Based Research Network. J. Dent..

[11] Zarone F., Ferrari M., Mangano F.G., Leone R., Sorrentino R. (2016). “Digitally oriented materials”: Focus on lithium disilicate ceramics. Int. J. Dent..

[12] Aboushelib M.N., Sleem D. (2014). Microtensile bond strength of lithium disilicate ceramics to resin adhesives. J. Adhes. Dent..

[13] Viskic J., Jokic D., Jakovljevic S., Bergman L., Ortolan S.M., Mestrovic S., Mehulic K. (2018). Scanning electron microscope comparative surface evaluation of glazed-lithium disilicate ceramics under different irradiation settings of Nd: YAG and Er: YAG lasers. Angle Orthod..

[14] Albakry M., Guazzato M., Swain M. (2003). Biaxial flexural strength, elastic moduli, and x-ray diffraction characterization of three pressable all-ceramic materials. J. Prosthet. Dent..

[15] Belli R., Geinzer E., Muschweck A., Petschelt A., Lohbauer U. (2014). Mechanical fatigue degradation of ceramics versus resin composites for dental restorations. Dent. Mater..

[16] Newman S.M., Dressler K.B., Grenadier M.R. (1984). Direct bonding of orthodontic brackets to esthetic restorative materials using a silane. Am. J. Orthod..

[17] Gillis I., Redlich M. (1998). The effect of different porcelain conditioning techniques on shear bond strength of stainless steel brackets. Am. J. Orthod. Dentofac. Orthop..

[18] Sabuncuoğlu F.A., Ertürk E. (2016). Shear bond strength of brackets bonded to porcelain surface: In vitro study. J. Istanb. Univ. Fac. Dent..

[19] Bayoumi R.E., El-Kabbany S.M., Gad N. (2019). Effect of Different Surface Treatment Modalities on Surface Roughness and Shear Bond Strength of Orthodontic Molar Tubes to Lithium Disilicate Ceramics. Egypt. Dent. J..

[20] Sundfeld Neto D., Naves L.Z., Costa A.R., Correr A.B., Consani S., Borges G.A., Correr-Sobrinho L. (2015). The effect of hydrofluoric acid concentration on the bond strength and morphology of the surface and interface of glass ceramics to a resin cement. Oper. Dent..

[21] Garboza C.S., Berger S., Guiraldo R.D., Fugolin A.P.P., Gonini-Júnior A., Moura S.K., Lopes M.B. (2016). Influence of Surface Treatments and Adhesive Systems on Lithium Disilicate Microshear Bond Strength. Braz. Dent. J..

[22] Al Rifaiy M.Q. (2018). Effect of Erbium-yttrium, scandium, gallium and garnet (Er-YSGG) laser on the bond strength of lithium disilicate ceramics. Pak. J. Med. Sci..

[23] Alqerban A. (2021). Lithium di silicate ceramic surface treated with Er, Cr: YSGG and other conditioning regimes bonded to orthodontic bracket. Saudi Dent. J..

[24] Souza K.B., Moura D.M.D., Silva S.E.G.D., Araújo G.M.D., Pinto R.D.A.S., Leite F.P.P., Özcan M. (2020). Effect of different surface treatments and multimode adhesive application on the Weibull characteristics, wettability, surface topography and adhesion to CAD/CAM lithium disilicate ceramic. J. Appl. Oral Sci..

[25] Mehmeti B., Kelmendi J., Iiljazi-Shahiqi D., Azizi B., Jakovljevic S., Haliti F., Anić-Milošević S. (2019). Comparison of shear bond strength orthodontic brackets bonded to zirconia and lithium disilicate crowns. Acta Stomatol. Croat..

[26] Labunet A., Kui A., Voina-Tonea A., Vigu A., Sava S. (2021). Orthodontic attachment adhesion to ceramic surfaces. Clin. Cosmet. Investig. Dent..

[27] Anadioti E., Aquilino S.A., Gratton D.G., Holloway J.A., Denry I., Thomas G.W., Qian F. (2014). 3D and 2D marginal fit of pressed and CAD/CAM lithium disilicate crowns made from digital and conventional impressions. J. Prosthodont..

[28] Ji M.-K., Park J.-H., Park S.-W., Yun K.-D., Oh G.-J., Lim H.-P. (2015). Evaluation of marginal fit of 2 CAD-CAM anatomic contour zirconia crown systems and lithium disilicate glass-ceramic crown. J. Adv. Prosthodont..

[29] Forster A., Ungvári K., Györgyey Á., Kukovecz Á., Turzó K., Nagy K. (2014). Human epithelial tissue culture study on restorative materials. J. Dent..

[30] Dilber E., Yavuz T., Kara H.B., Ozturk A.N. (2012). Comparison of the effects of surface treatments on roughness of two ceramic systems. Photomed. Laser Surg..

[31] Borges G.A., Sophr A.M., De Goes M.F., Sobrinho L.C., Chan D.C. (2003). Effect of etching and airborne particle abrasion on the microstructure of different dental ceramics. J. Prosthet. Dent..

[32] Kara H.B., Dilber E., Koc O., Ozturk A.N., Bulbul M. (2012). Effect of different surface treatments on roughness of IPS Empress 2 ceramic. Lasers Med. Sci..

[33] Ersu B., Yuzugullu B., Yazici A.R., Canay S. (2009). Surface roughness and bond strengths of glass-infiltrated alumina-ceramics prepared using various surface treatments. J. Dent..

[34] Blatz M.B., Sadan A., Kern M. (2003). Resin-ceramic bonding: A review of the literature. J. Prosthet. Dent..

[35] Kurtulmus-Yilmaz S., Cengiz E., Ongun S., Karakaya I. (2019). The effect of surface treatments on the mechanical and optical behaviors of CAD/CAM restorative materials. J. Prosthodont..

[36] Augusti D., Gabriele A., Francesca C., Dino R. (2015). Does sandblasting improve bond strength between nano-ceramic resin and two different luting composites. Bioceram. Dev. Appl..

[37] Keshvad A., Hakimaneh S.M.R. (2018). Microtensile bond strength of a resin cement to silica-based and Y-TZP ceramics using different surface treatments. J. Prosthodont..

[38] Chen J.H., Matsumura H., Atsuta M. (1998). Effect of etchant, etching period, and silane priming on bond strength to porcelain of composite resin. Oper. Dent..

[39] Prochnow C., Venturini A.B., Grasel R., Bottino M.C., Valandro L.F. (2017). Effect of etching with distinct hydrofluoric acid concentrations on the flexural strength of a lithium disilicate-based glass ceramic. J. Biomed. Mater. Res. Part B Appl. Biomater..

[40] Alao A.-R., Stoll R., Song X.-F., Abbott J.R., Zhang Y., Abduo J., Yin L. (2017). Fracture, roughness and phase transformation in CAD/CAM milling and subsequent surface treatments of lithium metasilicate/disilicate glass-ceramics. J. Mech. Behav. Biomed. Mater..

[41] de Kok P., Pereira G.K., Fraga S., de Jager N., Venturini A.B., Kleverlaan C.J. (2017). The effect of internal roughness and bonding on the fracture resistance and structural reliability of lithium disilicate ceramic. Dent. Mater..

[42] Sudré J.P., Salvio L.A., Baroudi K., Sotto-Maior B.S., Melo-Silva C.L., Assis N. (2020). Influence of surface treatment of lithium disilicate on roughness and bond strength. Int. J. Prosthodont..

[43] Reynolds I.R. (1975). A Review of Direct Orthodontic Bonding. Br. J. Orthod..

[44] Cardenas A.M., Siqueira F., Hass V., Malaquias P., Gutierrez M.F., Reis A., Perdigao J., Loguercio A. (2017). Effect of MDP-containing Silane and Adhesive Used Alone or in Combination on the Long-term Bond Strength and Chemical Interaction with Lithium Disilicate Ceramics. J. Adhes. Dent..

[45] Puppin-Rontani J., Sundfeld D., Puppin-Rontani R., Costa A., Correr A., Borges G., Sinhoreti M., Correr-Sobrinho L. (2016). HF concentration and etching times on lithium disilicate glass-ceramic. Dent. Mater..

[46] Kalavacharla V.K., Lawson N., Ramp L., Burgess J. (2015). Influence of etching protocol and silane treatment with a universal adhesive on lithium disilicate bond strength. Oper. Dent..

[47] Murillo-Gómez F., Rueggeberg F., De Goes M. (2017). Short- and Long-Term Bond Strength Between Resin Cement and Glass-Ceramic Using a Silane-Containing Universal Adhesive. Oper. Dent..

[48] Yao C., Zhou L., Yang H., Wang Y., Sun H., Guo J., Huang C. (2017). Effect of silane pretreatment on the immediate bonding of universal adhesives to computer-aided design/computer-aided manufacturing lithium disilicate glass ceramics. Eur. J. Oral Sci..

[49] Ankyu S., Nakamura K., Harada A., Hong G., Kanno T., Niwano Y., Örtengren U., Egusa H. (2016). Fatigue analysis of computer-aided design/computer-aided manufacturing resin-based composite vs. lithium disilicate glass-ceramic. Eur. J. Oral Sci..

[50] Lee H.-Y., Han G.-J., Chang J., Son H.-H. (2017). Bonding of the silane containing multi-mode universal adhesive for lithium disilicate ceramics. Restor. Dent. Endod..

[51] De Melo L.A., Moura I.D.S., De Almeida E.O., Junior A.C.F., Dias T.G.D.S., Leite F.P.P. (2019). Efficacy of prostheses bonding using silane incorporated to universal adhesives or applied separately: A systematic review. J. Indian Prosthodont. Soc..

[52] Chen L., Shen H., Suh B.I. (2013). Effect of incorporating BisGMA resin on the bonding properties of silane and zirconia primers. J. Prosthet. Dent..

[53] Kim Y.-R., Kim J.-H., Son S.-A., Park J.-K. (2021). Effect of Silane-Containing Universal Adhesives on the Bonding Strength of Lithium Disilicate. Materials.

[54] Dasy H., Dasy A., Asatrian G., Rózsa N., Lee H.F., Kwak J.H. (2015). Effects of variable attachment shapes and aligner material on aligner retention. Angle Orthod..

